# A compact single layer dual-polarized antenna with wideband operation, high isolation, and high front-to-back ratio for 5G applications

**DOI:** 10.1038/s41598-025-08008-z

**Published:** 2025-07-15

**Authors:** Niamat Hussain, Hung Tran, Dat Tran-Huy, Phuong Kim-Thi

**Affiliations:** 1 Electronics and Nanoscale Engineering, University of Glasgow, Glasgow, UK; 2 Department of Convergence Engineering for Intelligent Drone, Sejong University, Seoul, 13391 Republic of Korea; 3 Faculty of Electrical and Electronic Engineering, PHENIKAA University, Hanoi, 12116 Vietnam; 4 Faculty of Electrical and Electronics Engineering, Thuyloi University, Hanoi, Vietnam

**Keywords:** Electrical and electronic engineering, Mathematics and computing

## Abstract

This paper proposed a simple design of dual-polarized antenna with a single layer and compact structure for 5G applications. The proposed antenna performs several advantages of wideband, high isolation, as well as high front-to-back ratio characteristics. Instead of using complicated feeding network or stacked structure, a directed-fed crossed patch antenna is employed. Noted that by locating the feeding locations close to the center, high isolation can be achieved without requiring any complicated feeding scheme. Additionally, four parasitic patches are introduced to not only enhance the operating bandwidth but also improve the front-to-back ratio. The final antenna with compact overall dimensions of 0.46$\lambda$ $\times$ 0.46$\lambda$ $\times$ 0.02$\lambda$ exhibits wideband operation of 7.5% (4.76–5.13 GHz) and high isolation of better than 23 dB. Besides, despite having compact size, the antenna achieves a broadside gain of about 6.0 dB and the front-to-back ratios of around 15 dB across the operating bandwidth.

## Introduction

Dual-polarized antennas have been widely used in wireless communication systems due to their capabilities of increasing channel capacity and decreasing the effect of multipath interference^1^. Additionally, antennas with compact size and low complexity are preferred as they can easily integrated into wireless devices.

Microstrip patch antenna has been used as an effective solution to design dual-polarized antenna with compact size features. Various methods have been published to increase the port-to-port isolation of this antenna type. The first approach employs differential feeding networks, such as hybrid coupler, T-junction or Wilkinson power divider^2–8^. These antennas are commonly designed with multiple layers and complicated feeding networks. Although wideband and high isolation can be achieved, these improvements are made at the expense of profile, bandwidth, gain, as well as structure complexity. For the compactness and simplicity purposes, a configuration of patch antenna with capacitive and aperture-coupled feeds is one of the effective solutions. In^9–11^, the patches are capacitively fed with introduced airgap, leading to bulky structures and high profile of about 0.07$\lambda$. In^12,13^, the patches are separately excited by feeding lines through slots etched in ground planes. Despite achieving lower profile, multi-layer structure and high back-lobe radiation due to the unwanted radiation from the slots are their disadvantages. Besides, high isolation is obtained at the cost of complexity and antenna size.

Only a few dual-polarized patch antennas with compact size and simple feeding scheme characteristics have been reported. The patches can be directly probe-feed^14^ or corner-feed by microstrip lines^15^. Meanwhile, the patches in^16,17^ are loaded with more capacitance through fence-strip resonator or fence to achieve compact size and high isolation. Alternatively, the dual-polarized patch is designed with L-shaped short pins, slots etched on the ground can obtain high isolation with small footprint and low profile^18^. Overall, although the designs^14–18^ exhibit high isolation, they suffer from several critical drawbacks of narrow operating bandwidth, complicated and high-profile structures.

In this paper, a simple design of single-layer compact dual-polarized antenna is proposed. The dual polarization diversity with high isolation is based on a direct-fed crossed patch antenna. Meanwhile, the operating bandwidth and the front-to-back ratio can be improved with the aid of four parasitic elements. The proposed antenna exhibits high isolation of more than 23 dB across the operating bandwidth of 7.5%. Additionally, despite having compact structure, a high front-to-back ratio around 15 dB can be achieved.

## Dual-polarized crossed patch antenna

**Fig. 1:**
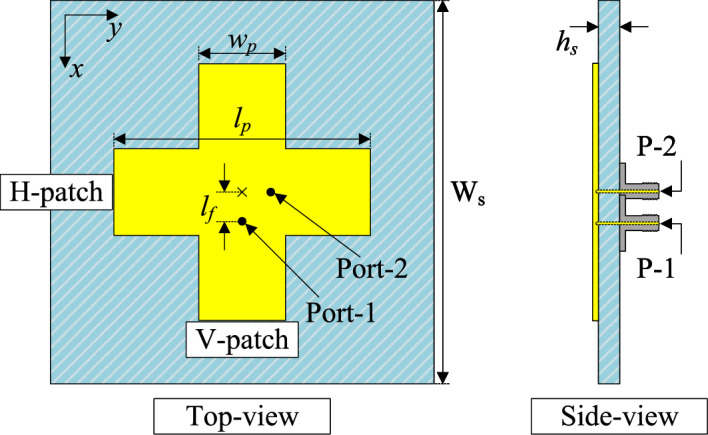
Geometry of the dual-polarized crossed patch antenna.

Figure 1 shows the geometrical configuration in terms of top- and side-view of the proposed dual-polarized crossed patch antenna. The crossed patch is a combination of two half-wavelength rectangular patches, vertical patch (V-patch) and horizontal patch (H-patch). These patches have similar dimensions of $L_p$ and $W_p$, and they are designed on a single-layer Taconic RF-35 substrate with dielectric constant of 3.5 and copper metal material. Two 50-$\Omega$ coaxial cables are employed to directly excite the patch at two different positions, Port-1 and Port-2. The optimal dimensions are as follows: $W_s = 27$, $l_p = 18$, $w_p = 6$, $l_f = 2$, $h_s = 1.52$ (unit: mm).

**Fig. 2:**
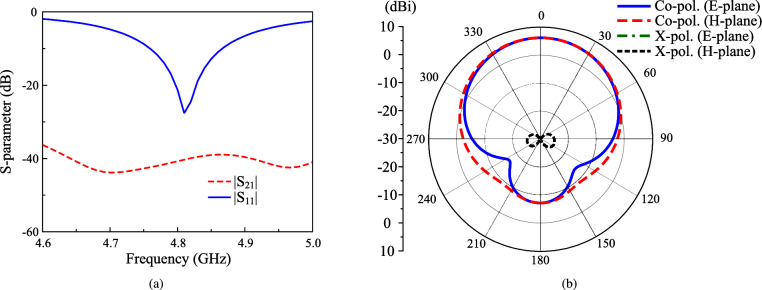
Simulated (**a**) S-parameter and (**b**) radiation patterns at 4.8 GHz of the proposed dual-polarized crossed patch antenna.

The simulated scattering parameter (S-parameter) of the proposed dual-polarized antenna is shown in Fig. 2. The simulated reflection coefficient of less than –10 dB ranges from 4.76 to 4.86 GHz (2.1%). Meanwhile, the inter-port isolation is about 40 dB within the impedance matching bandwidth. In terms of gain, the main beam has a broadside direction with gain of about 6.2 dBi. Meanwhile, using a small ground plane to achieve compact size leads to high back-lobe radiation of about –7.2 dB.

**Fig. 3:**
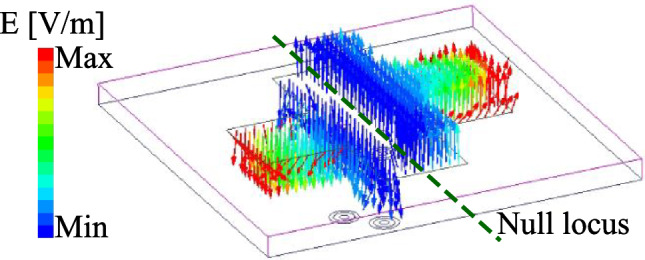
Simulated E-field distribution on the crossed patch.

When placing two probe-fed patches in perpendicular configuration, two dominant orthogonal modes, i.e., $\hbox {TM}_{10}$ and $\hbox {TM}_{01}$, are the fundamental operating modes for these patches. Assume that the V-patch is fed, voltage distribution along this patch will be zero in the center, maximum on one edge (positive charge) and minimum on the opposite edge (negative). A null locus with zero potential appears along the equator that can be considered as a virtual ac ground plane. Whatever the feeding position lies along this line, including the horizontal dipole, experiences no coupling from the vertical dipole. For verification, Fig. 3 shows the simulated electric field (E-field) distribution at 4.8 GHz on the proposed dual-polarized antenna when the V-patch is excited. As observed, the null locus appears at the center and thus, the cross coupling between the V-patch and H-patch can be suppressed. It also brings to extremely low cross-polarization of about –42 dB, shown in Fig. 2b.

## Dual-polarized wideband antenna

**Fig. 4:**
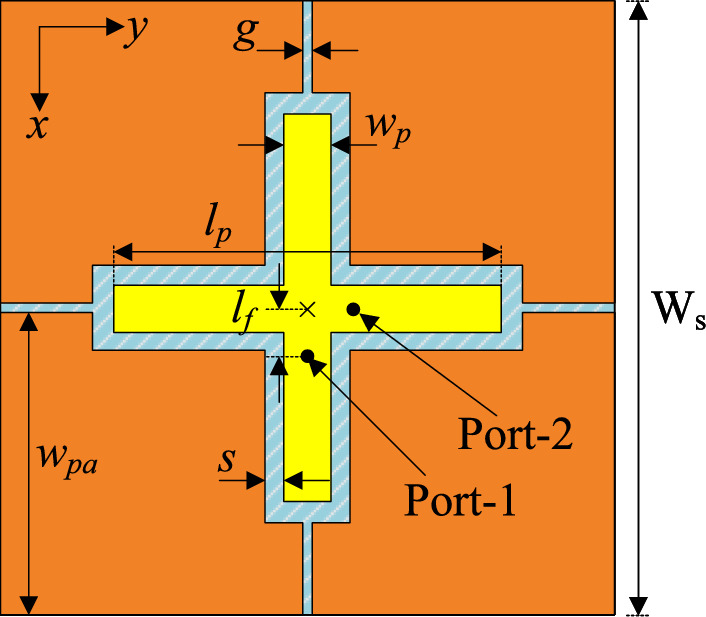
Geometry of the proposed wideband dual-polarized antenna.

As the dual-polarized antenna discussed in Section 2 exhibits narrow bandwidth, four parasitic elements are positioned in proximity to the crossed patch. Figure 4 shows the geometry of the proposed wideband dual-polarized antenna. The optimal design parameters are as follows: $W_s = 27$, $l_p = 17.6$, $w_p = 2$, $l_f = 2$, $h_s = 1.52$, $s = 1$, $g = 0.15$, $w_{pa} = 13.4$ (unit: mm).

**Fig. 5:**
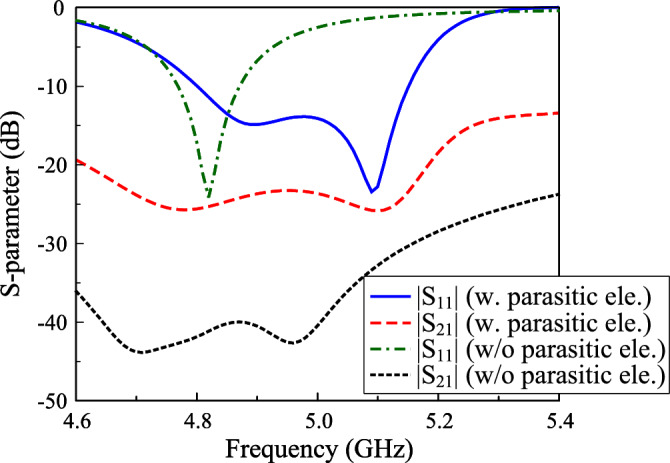
Simulated S-parameter of the proposed wideband dual-polarized antenna.

**Fig. 6:**
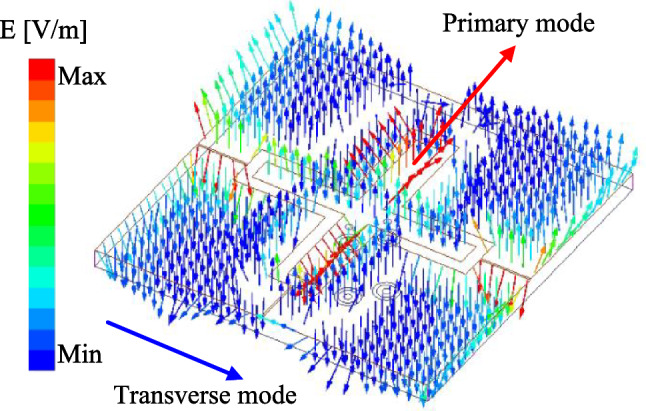
Simulated E-field distribution of the proposed wideband dual-polarized antenna.

The simulated S-parameter and radiation patterns at 4.9 GHz of the proposed design is presented in Fig. 5. In comparison with the reference antenna in the previous section, the impedance bandwidth is increased from 2.1% (4.76–4.86 GHz) to 7.2% (4.80–5.16 GHz). Here, two resonances in the $|S_{11}|$ profile are obtained with the lower one (4.9 GHz) produced by the crossed patch and the other (5.1 GHz) produced by the parasitic elements. However, the disturbance caused by the parasitic elements supports the transverse mode depicted in Fig. 6, which significantly affects isolation of the antenna. The isolation within the impedance bandwidth of the antenna with parasitic elements is better than 22 dB.

**Fig. 7:**
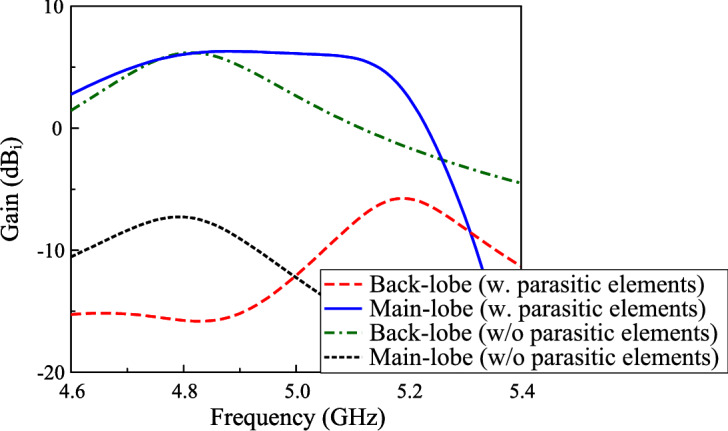
Simulated realized gain of main-lobe and back-lobe for different antenna configurations.

Besides contributing to improving the operating bandwidth, the parasitic elements also have significant effect on the back-lobe radiation of the antenna. Figure 7 shows the realized gains of the main-lobe and back-lobe radiation. As observed, the main-lobe levels of both antennas are quite similar in their operating bands. Meanwhile, the back-lobe levels observe a significant difference. In the frequency range of lower than 5.0 GHz, the antenna with parasitic elements has smaller back radiation. As this range radiated by the crossed patch, the power tends to couple with the parasitic elements rather than diffraction at the edges of the substrate as the antenna without parasitic elements (depicted in Fig. 1). Higher frequency range produced by the parasitic elements, whose outer boundaries are similar to the ground plane. Accordingly, high diffractions will occur, leading to high back radiation. For performance improvement, one possible solution is to expand the ground plane, though this comes at the cost of increased overall antenna dimensions. Alternatively, substrate integrated waveguide (SIW) cavities offer an effective solution without significantly enlarging the antenna size.

## Key parameter studies

**Fig. 8:**
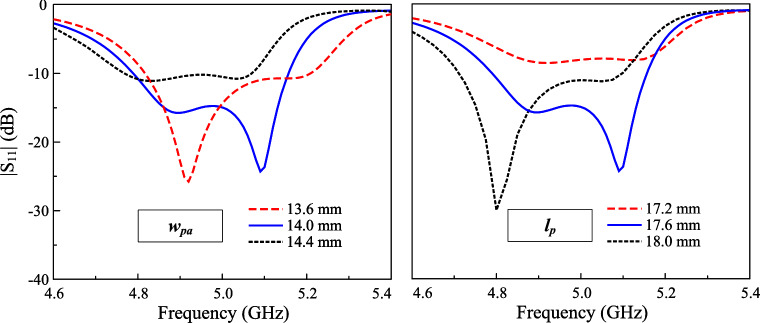
Simulated $|S_{11}|$ for different sizes of parasitic elements ($w_{pa}$) and crossed patch ($l_p$).

**Fig. 9:**
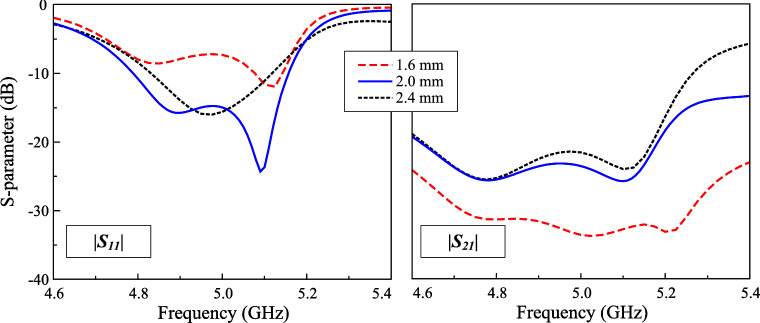
Simulated $|S_{11}|$ and $|S_{21}|$ for different feeding positions, $l_f$.

**Fig. 10:**
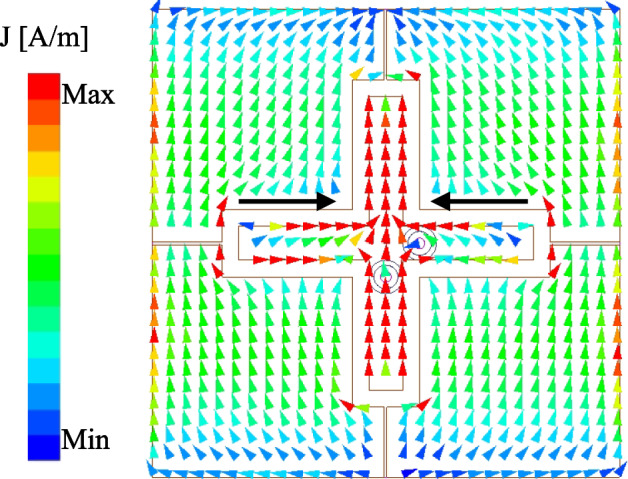
Simulated current distribution at 4.9 GHz with Port-1 excitation.

**Fig. 11:**
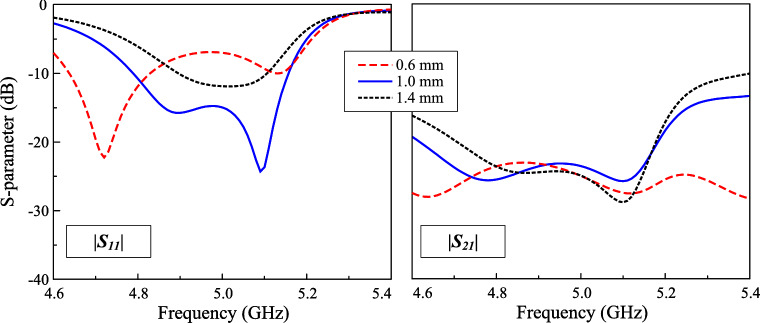
Simulated $|S_{11}|$ and $|S_{21}|$ for different gaps, *s*.

This Section presents several key parameters, which are critical to determine the operating performance of the proposed antenna. Noted that when one parameter is studied, the others are kept at the optimal values. The first important parameter is the size of the parasitic elements, $w_{pa}$. As these parasitic elements are utilized to produce additional resonance in the high frequency range, changing $w_{pa}$ has significant effect on the higher frequency, as demonstrated in Fig. 8. Larger $w_{pa}$. makes the higher resonance in the $|S_{11}|$ profile shift towards lower frequency range. Similarly, the effect when changing the length of the crossed patch, $l_p$, on the operating frequency of the lower resonance in the $|S_{11}|$ profile can be observed. Finally, the feeding position, $l_f$, is considered as shown in Fig. 9. This parameter strongly determines the matching and isolation performance of the proposed design. It can be seen obvious that when the feeding position close to the center, the antenna performs poor impedance but better isolation. The reason behind the isolation improvement when the feeding position is close to the center can be explained based on Fig. 10, which shows the current distribution on the crossed patch at 4.9 and 5.1 GHz with Port-1 excitation (V-patch). In both frequencies, the current flowing on the H-patch is in opposite directions. The magnitude of the current flowing on the H-patch will close to 0 when the feeding position moves closer to the center due to the cancellation of the out-of-phase currents. Moving the feeding position far away from the center will increase the degree of symmetry, leading to worse isolation. Here, the feeding position is chosen at the compromise between the matching and isolation performance. Finally, the impact of the gap between the crossed patch and the parasitic elements, *s*, on antenna performance is illustrated in Fig. 11. It is evident that *s* has a minimal influence on the transmission coefficient. In contrast, the reflection coefficient is significantly affected. Changing the gap will affect the capacitance formed by the crossed patch and the parasitic elements, leading to a shift of the resonant frequency, particularly noticeable in the lower frequency band.

**Fig. 12:**
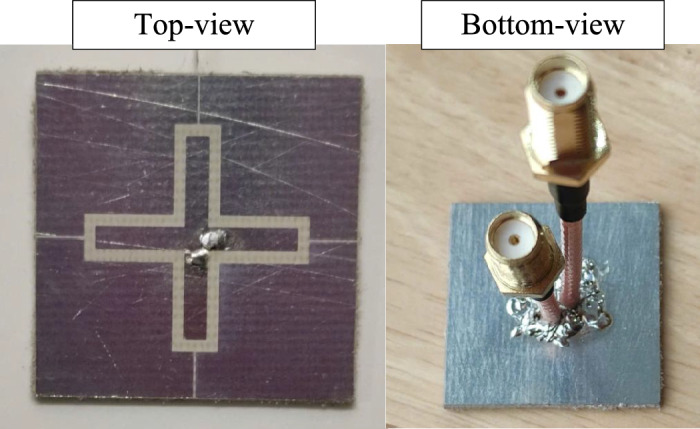
Photographs of the fabricated antenna prototype.

**Fig. 13:**
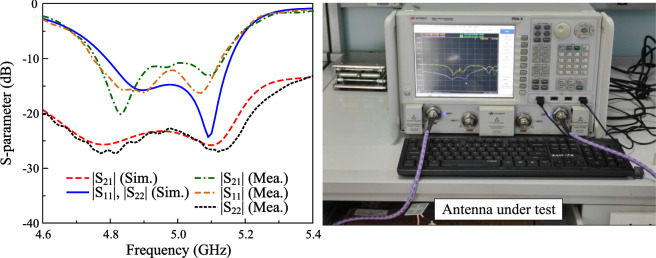
Simulated and measured S-parameter of the proposed antenna.

## Measured results

For validation, an antenna prototype is fabricated as shown in Fig. 12 and measurement. The simulated and measured S-parameter results of the proposed antenna are depicted in Fig. 13. Here, the common operating bandwidth for both reflection coefficients ($|S_{11}|$ and $|S_{22}|$) lower than –10 dB is from 4.76 to 5.13 GHz, corresponding to about 7.5%. Across this band, isolation is always better than 23 dB.

**Fig. 14:**
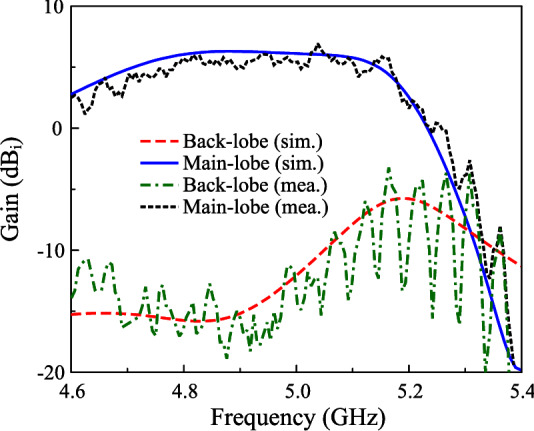
Simulated and measured gain results of the proposed antenna.

**Fig. 15:**
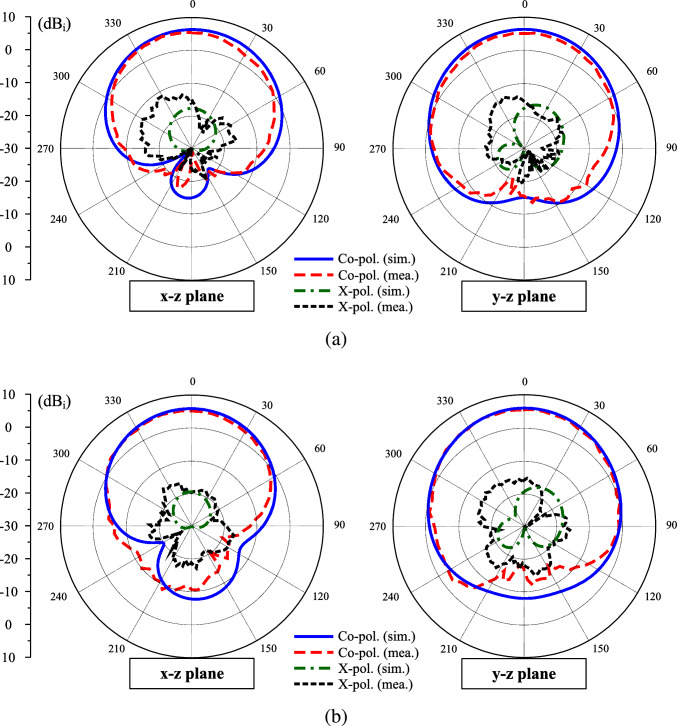
Gain radiation patterns of the proposed antenna at (**a**) 4.9 and (**b**) 5.1 GHz.

In terms of far-field performance, the measured data with Port-1 excitation is presented for brevity. Figure 14 shows the simulated and measured gain in the forward and backward directions. As observed, the simulated and measured data are well matched. The antenna exhibits broadside gain around 6.0 dBi and the front-to-back ratio around 15 dB within the operating bandwidth. The gain radiation patterns at 4.9 and 5.1 GHz are plotted in Fig. 15. The antenna has quite symmetrical radiation patterns around the broadside direction. Meanwhile, the polarization isolation in the main direction is better than 20 dB.

The advantages of the proposed design in comparison with the related works can be demonstrated in Table 1. Overall, it is obvious that the proposed antenna has very compact size with simple structure configuration (no vias, no airgap, single layer), while achieving comparable operating performance. Wide bandwidth can be attained in^3,5,12^, but bulky and complicated structures are the critical drawbacks. In comparison with the directed feed designs^16–18^, the proposed antenna has smaller size, wider bandwidth, higher gain and better FBR.

**Table 1:** Performance comparison among dual-polarized patch antennas.

References	Overall size ($\lambda$)	No. of layers	Feeding scheme	Airgap/Vias	BW (%)	Isolation (dB)	Max. Gain (dBi)	Avg. FBR (dB)
^3^	2.06 $\times$ 2.06 $\times$ 0.24	5	Differential	Y / N	45	$\ge$ 35	10.5	22
^5^	0.72 $\times$ 0.72 $\times$ 0.12	5	Differential	Y / N	24	$\ge$ 30	8.7	Not given
^10^	1.82 $\times$ 0.82 $\times$ 0.06	3	Capacitive	N / N	5.7	$\ge$ 25	8.3	22
^12^	2.81 $\times$ 2.81 $\times$ 0.06	1	Slot	N / Y	30	$\ge$ 40	8	22
^16^	1.23 $\times$ 1.23 $\times$ 0.04	2	Directed	N / Y	4.9	$\ge$ 20	4.9	12
^17^	1.43 $\times$ 1.43 $\times$ 0.07	3	Directed	Y / Y	3.2	$\ge$ 32	5.6	20
^18^	0.35 $\times$ 0.35 $\times$ 0.04	2	Directed	N / Y	3.1	$\ge$ 35	4.1	8
Prop.	0.46 $\times$ 0.46 $\times$ 0.02	1	Directed	N / N	7.5	$\ge$ 23	7	15

## Conclusion

A simple design of compact and single-layer dual-polarized antenna has been investigated and presented in this paper. The proposed antenna is based on the crossed patch and four parasitic patches, which are utilized for bandwidth and front-to-back ratio enhancements. The final antenna has compact dimensions of 0.46$\lambda$ $\times$ 0.46$\lambda$ $\times$ 0.02$\lambda$. It can exhibit a wideband performance of 7.5% with high isolation of better than 23 dB and gain around 6.0 dBi. In comparison with the related works, the proposed antenna has advantages of simple and compact structures, while it can perform comparable operation characteristics.

## Data Availability

Data is provided within the manuscript.
